# ‘*Patients are left not knowing*’: Trastuzumab (Herceptin) cardiac risk information and management in HER2-positive breast cancer—a qualitative study of patient experience

**DOI:** 10.1007/s00520-026-10357-y

**Published:** 2026-02-05

**Authors:** Karina Dolgilevica, Bethany Chapman, Nazanin Derakshan

**Affiliations:** 1 School of Psychological Sciences, Birkbeck University of London, London, UK; 2 School of Psychology and Clinical Language Sciences, University of Reading, Reading, UK; 3 National Centre for Integrative Oncology (NCIO), Reading, UK

**Keywords:** Breast cancer, Herceptin, Qualitative method, Patient experience, Cardiac dysfunction

## Abstract

**Purpose:**

The current study investigated the extent of knowledge and care regimen regarding the possible risks of cardiac dysfunction from Trastuzumab (Herceptin) treatment in women with either a diagnosis of primary or metastatic breast cancer who had undergone or were receiving Trastuzumab at the time of the study.

**Method:**

In a qualitative interview, participants were asked questions regarding awareness of cardiac risks of Trastuzumab therapy and knowledge of cardiac damage symptoms, as well as questions concerning the regimen of heart function monitoring during and beyond their course of treatment. Thematic analysis was used to analyse the data.

**Results:**

The emerging themes from the thematic analysis revealed inconsistencies in (a) knowledge and awareness of cardiac damage risk prior to taking Trastuzumab, (b) monitoring of cardiac function and risks of cardiac dysfunction during and beyond treatment, and (c) awareness of signs of possible cardiac damage of Trastuzumab during treatment.

**Conclusions:**

Our results suggest that patients feel unsupported and ill-informed about the possible risks of cardiac damage from Trastuzumab. This has implications for the self-management of symptoms and mitigation of risks for cardiac damage and suggests the need for improvement in patient care.

## Introduction

Breast cancer is the most prevalent cancer in the UK [1] with every 10 min a woman being diagnosed [2]. Approximately 20% of breast cancer tumours are responsive to the HER2 epidermal growth factor [3]. HER2-positive breast cancers are usually of an aggressive nature with a higher likelihood of recurrence [3]. Trastuzumab, commonly known by its main brand name ‘Herceptin’, is considered the ‘wonder drug’ of HER2-positive breast cancer. It is a drug treatment that works by inhibiting the growth of the HER2 gene which increases epidermal growth malignancy. The efficacy of Trastuzumab in treating breast cancer and delaying or preventing breast cancer recurrence in patients diagnosed with the HER2-responsive protein has been substantiated [4].

With the advent of Trastuzumab, survival outcomes have increased for women with a primary or metastatic diagnosis of HER2-positive breast cancer [5]. Trastuzumab is usually taken either on its own or in combination with chemotherapy [6–8]. It is expected that, on average, HER2-positive patients will receive Trastuzumab via an injection over three-week periods for a length of 18 months, although other forms of drug intake and periods are possible [9].

Whilst Trastuzumab is regarded as an effective drug, numerous studies have highlighted its side effects, which adversely impair quality of life during treatment and beyond [10]. These include but are not limited to haematological toxicity, especially when administered with chemotherapy [11] and cardiac damage, including cardiac death [12]. Other symptoms involving changes to the skin and nail have also been noted, as well as fatigue, pain, nausea, and peripheral neuropathy, to name a few [10].

The adverse effects of Trastuzumab on cardiac function are of particular interest as evidence points to an increased risk of cardiotoxicity [13]. Collective evidence suggests an increased risk of congestive heart failure and decreased left ventricular ejection fraction is associated with the use of Trastuzumab in individuals with metastatic breast cancer [12, 14]. Similar findings have been reported in women with primary breast cancer [11]. The risks of cardiac dysfunction in the left ventricular ejection fraction have been reported to be up to 7% when using Trastuzumab alone and up to 27% when using Trastuzumab alongside other chemotherapy treatments [15, 16]. Decreased left ventricular ejection fraction is a risk factor for congestive heart failure. It is strongly recommended that patients are routinely monitored for cardiac function using electrocardiogram prior to starting Trastuzumab and during treatment so that treatment plans can be adjusted should cardiac dysfunction be apparent [12]. As Trastuzumab-related cardiotoxicity is mainly seen at the earlier stages of treatment, it is imperative that patients are monitored early [12] and at regular intervals whilst receiving treatment and every 6 months for at least 2 years post discontinuation of Trastuzumab in patients with pre-existing cardiac problems [17].

The potential damage to cardiac health has implications for patients as well as clinicians as they naturally face difficult decisions when balancing the risks and benefits of Trastuzumab for patient use [10]. Usually, the benefits of Trastuzumab are emphasised, and the research supporting and quantifying the benefits of this drug is plentiful. An existing gap is the lack of studies quantifying the possible adverse risks to cardiac health and malfunction compared to the wealth of studies highlighting the benefits of Trastuzumab. This has led to recent studies raising concern regarding the level of drug toxicity, which can put patients at risk of damage to cardiac health [10, 12, 13].

Whilst research is emphasising the need to highlight the risks of Trastuzumab on cardiac damage, there is no qualitative study which has investigated patient knowledge and understanding of Trastuzumab effects on cardiac health. This gap needs addressing as patients should be able to weigh the costs and benefits of therapies such as Trastuzumab. Awareness of risks and side effects of drug treatment can help empower patients in their decision-making and aid in patient-doctor communication and trust. Using a qualitative interview approach, we investigated patient knowledge of the risks and benefits of Trastuzumab regarding damage to cardiac health in women who had undergone Trastuzumab treatment for primary or metastatic breast cancer. Participants were also asked questions about heart function monitoring and awareness of symptoms of cardiac damage.

## Methods

The design was a qualitative interview study. The study was approved by the School of Psychology and Clinical Language Science Ethics Research Committee at the University of Reading (Reference number: ND-2023-021-ND) and performed in accordance with the Declaration of Helsinki.

## Participants

Women with a diagnosis of breast cancer living in the United Kingdom (UK) were recruited using purposive sampling via advertisements placed on social media platforms such as Facebook. The advert was displayed on public and private support networks including ‘Building Resilience in Breast Cancer Centre’ (BRiC, https://briccentre.co.uk/). Following the guidelines from Braun & Clarke (2013), we planned to recruit eight women; however, due to the high interest from women wanting to discuss their experience with Trastuzumab treatment, a total of 14 women were interviewed.

Inclusion criteria included: (1) aged 18 or over, (2) diagnosis of primary or secondary breast cancer (stage I–IV), (3) must have received or could still be receiving Trastuzumab treatment, and (4) must be post-active chemotherapy and/or radiotherapy treatment for primary breast cancer.

## Procedure

After registering an interest in the study via email, participants were sent the study information and participant inclusion criteria to confirm their eligibility. Following confirmation, a secure URL link to access the online consent form and demographics questionnaire programmed on Gorilla Experimenter Builder (www.gorilla.sc) was sent in addition to a list of available interview appointments. Participants were asked to provide online consent to take part in the study, as well as verbal consent at the start of the telephone interview to confirm that the interviewer (BC) could audio-record their discussion. Participants were informed at the start of the interview that the information provided would be used for research purposes only and any identifiable information would be removed from the final transcript to ensure their anonymity. Further, they were given an opportunity to ask any questions or discuss any possible concerns. Interviews were conducted via MS Teams (https://www.microsoft.com/en-gb/microsoft-teams) calls (cameras off), with only the researcher and participant present. Interviews lasted on average 26.9 min.

All clinical information relating to breast cancer diagnosis was self-reported and recorded using the demographic questionnaire. Following the interview, women received a £7 e-gift voucher to compensate for their time.

## Materials

### Demographic questionnaire

The demographic questionnaire (developed by authors) consisted of 24 questions probing participants’ sociodemographic (e.g. age, ethnicity, and education), breast cancer (e.g. stage and treatment), diagnosis of anxiety or depression (e.g. current or prior diagnosis), and work-related characteristics (e.g. job role).

### Interview schedule

The semi-structured interview topic guide was developed by our multi-disciplinary team of researchers including a cardiologist using their expertise and knowledge of cardiac dysfunction and Trastuzumab to explore women’s experiences with this treatment. After the initial interview schedule was devised, it was reviewed and refined by BC and ND (both female researchers), with a few minor changes. The interview guide included a series of questions relating to the role of cardiac risk and the decision to proceed with Trastuzumab therapy, awareness of risks of cardiac damage from Trastuzumab, as well as about the regimen of heart scans (or monitoring) received during treatment. Women were defined as the experts in their experience and any new topics introduced by them were fully explored. No changes were made to the interview schedule during data collection.

## Data analysis

Interviews were conducted and audio-recorded using MS Teams and then transcribed verbatim. Grammatical errors were maintained to reflect the woman’s voice. Transcripts were then anonymised using a pseudonym ID. The stages of analysis followed the phases of qualitative thematic analysis [18, 19]: (1) data familiarisation; (2) initial codes (open coding); (3) formation of themes; (4) reviewing themes; (5) defining and naming themes; and (6) producing the report. A bottom-up approach was applied, and themes were identified within their explicit meaning (e.g. we were not looking for meaning beyond what the women described). The interviews were independently analysed and coded by KD and supervisor ND. There were only minor differences in themes which were resolved by mutual agreement. To mitigate potential bias and enhance trustworthiness, KD and ND regularly discussed methodological decisions and interpretations throughout all phases of analysis and write-up. NVivo Pro 12 software (QSR International Pty Ltd., 2018) was utilised to facilitate the initial coding and analysis process, as well as support secure storage. Saturation was reached before the 14th interview. Interviews were not returned to participants for review or feedback due to the sensitive nature of the research. Illustrative quotes are provided in our results to support the findings.

## Results

### Sample characteristics

Table 1 shows the sociodemographic, clinical, and work-related characteristics of the 14 women recruited to take part in the interview.

**Table 1:** Participant sociodemographic, clinical, and work characteristics

	***n* = *14 (%)***
**Sociodemographic**	
Age	Mean = 51.4 (range 40.0–64.0)
Education	
Secondary education	2 (14.3)
Further education	2 (14.3)
Higher education	10 (71.4)
Ethnicity	
English/Welsh/Scottish/NI/British	12 (85.7)
Irish	1 (7.1)
Any other white background	1 (7.1)
**Work**	
Employment type	
Paid work	6 (42.9)
Unpaid work	1 (7.1)
Not currently working	4 (28.6)
Other	3 (21.4)
**Clinical history**	
Age at primary diagnosis	Mean = 44.2 (range 36.0–53.0)
Age at secondary diagnosis	Mean = 46.2 (range 39.0–51.0)
Stage	
2	3 (21.4)
3	1 (7.1)
4	5 (35.7)
I don’t know	5 (35.7)
Diagnosis	
Ductal carcinoma in situ (DCIS)	3 (21.4)
Inflammatory breast cancer (IBC)	4 (28.6)
Mixed DCIS and IBC	6 (42.9)
Invasive ductal carcinoma	1 (7.1)
Active treatment	
Yes	5 (35.7)
No	9 (64.3)
Current endocrine therapy	
Yes	12 (85.7)
No	2 (14.3)
Current Herceptin therapy	
Yes	7 (50.0)
No	7 (50.0)
Duration of Trastuzumab therapy (months)	Mean = 24.5 (range 9.0–96.0)
Diagnosis of anxiety and/or depression PRIOR to diagnosis of breast cancer	2 (14.3)
Current diagnosis of anxiety and/or depression	1 (7.1)

Three main themes with nine subthemes emerged from the interviews. The following main themes were identified: (1) ‘The role of cardiac damage risk on women’s decision to proceed with Trastuzumab treatment’, (2) ‘Monitoring of cardiac function and risks during treatment and beyond’, and (3) ‘Knowledge on cardiac damage risks of Trastuzumab’ (please refer to Table 2 for a full list of themes and subthemes).

**Table 2:** Main themes and subthemes from qualitative analysis

Main themes	Subthemes
***(1) The role of cardiac damage risk on women’s decision to proceed with Trastuzumab treatment***	*Cancer recurrence risk outweighs cardiac damage risk*
	*Reassured by regular cardiac monitoring*
***(2) Monitoring of cardiac function and risks during treatment and beyond***	*Professional monitoring during treatment*
	*Cardiac imaging regimen*
	*Inadequate long-term cardiac health monitoring*
***(3) Knowledge on cardiac damage risks of Trastuzumab***	*Overwhelmed with information*
	*Unaware of cardiac damage signs*
	*Ill-informed about cardiac damage risks*
	*Involve patients to improve patient experience*

### Theme 1: The role of cardiac damage risk on women’s decision to proceed with Trastuzumab treatment

#### Cancer recurrence outweighs cardiac damage risk

Overall, women reported that potential cardiac damage (CD) risk had little to no impact on their decision to have Trastuzumab (Herceptin) as part of their treatment. Most women stated that they would agree to continue with Trastuzumab treatment even if CD risk was high:

*‘It made zero impact on my decision.’ [Interview 4]*

*‘…it’s a small price to pay if it gives me life.’ [Interview 13]*

Women generally expressed a very positive view of Trastuzumab. Some described it as the ‘wonder drug’, saying they felt ‘lucky’ to have it and the possibility of not having access to it being ‘devastating’. All women said that the priority of treating the cancer outweighed the risks of any side-effects, including cardiac damage:

*‘Oh, it’s the wonder drug. It was like there was no question I was gonna have it, you know, in my mind, it was like you've gotta have this.’ [Interview 8]*

*‘…because I know that really was the big thing of saving my life …it was in my mind, you deal with the collateral damage afterwards kind of thing.’ [Interview 2]*

Despite the potential risks of CD, preventing cancer recurrence remained a top priority even among those with personal or family history of cardiovascular disease and those who developed cardiac complications during Trastuzumab treatment. Here is how one woman who experienced heart failure during her Trastuzumab treatment described how her anxiety about cancer recurrence motivated her decision to resume treatment:

*‘… even with all the knowledge I’ve got, I’ve still made that choice to go on to it because the anxiety of the cancer is…is worse than the heart, which you know, even though my oncologist just said to me it could be the heart that finishes you off before the cancer...’ [Interview 3]*

For many, being eligible to receive Trastuzumab was a strong ‘selling point’ motivating their treatment choices and decisions:

*‘So, I’d actually have the chemotherapy knowing it would get me more Herceptin.’ [Interview 12]*

*‘Initially I was like I just don’t want chemo… and they were like if you don’t have chemo, you can’t have Herceptin and Herceptin is the wonder drug.’ [Interview 8]*

Only one woman who experienced a recent cancer recurrence began reconsidering her long-term treatment plan after learning about Trastuzumab CD risks, wondering whether it is giving her any ‘extra protection’.

#### Reassured by regular cardiac monitoring

Despite willing to accept ‘any’ risks, some women said that if they knew that their risk of CD was lower it would have made their decision to proceed with Trastuzumab somewhat ‘easier’. Most have said they felt ‘reassured’ by knowing that their heart function will be monitored regularly using heart imaging:

*‘… with monitoring it feels like it is a fairly safe drug to take as long as you go for your regular echoes.’ [Interview 5]*

Some women also felt reassured by knowing that cardiac damage can be managed using medication should any damage occur. Overall, most women were informed that in case of decline to heart function, a mid-treatment recovery break may be necessary. Whilst feeling reassured, the women were generally driven to stay on or to complete their treatment with some feeling very ‘anxious’ or devastated about the prospect of treatment breaks:

*‘… I had to put the date on the calendar that I was finishing treatment and that was the date I was working to. Um, I think I would have been gutted if-if I’d not completed it.’ [Interview 11]*

### Theme 2: Monitoring of cardiac function and risks during treatment and beyond

#### Professional monitoring during treatment

Generally, women reported that most of the clinical contact during their Trastuzumab treatment was with their oncologist and with nurses who administered Trastuzumab. Some of the women were referred to cardiology due to developing some cardiac damage during their treatment, but the vast majority had not seen a cardiologist throughout their treatment and had limited awareness of the extent of cardiology input during their treatment. They said that the decision to stop Trastuzumab or heart scan results were typically communicated through their oncologist. Some women felt ‘cut out’ due to a lack of direct communication with cardiology professionals:

*‘I’ve never had a letter back from that on that cardiologist. Nothing. It’s almost like they cut out me as the middle person…’ [Interview 3]*

*‘… they knew about me being under a cardiologist um, and I can’t remember but, but hopefully they would have contacted them to see if there are any reasons why I shouldn’t have Herceptin.’ [Interview 7]*

One woman with a cardiac history pre-breast cancer diagnosis spoke about feeling ‘lucky’ to have access to cardiology and tighter multidisciplinary support throughout her Trastuzumab treatment because of her history. In contrast, another woman experienced a 7-month interruption in Trastuzumab treatment due to poor communication with cardiology specialists, leaving both her and her oncologist expecting further input and ultimately feeling ‘let down’ by the cardiology service.

#### Cardiac imaging regimen

Most women reported undergoing regular 3-monthly echocardiograms during their Trastuzumab treatment period; some were scanned on a 6 monthly basis if they were a stable secondary diagnosis case. The women generally found the imaging appointments were easily accessible and straightforward. Two women experienced physical challenges, finding some of their scans painful when applied to the mastectomy surgery site. Aside from physical challenges, a couple of other women reported irregular imaging, highlighting that such irregularity can lead to unnecessary worry and uncertainty. For example, one woman on an 18-round Herceptin plan received only two heart scans, which made her feel concerned about her heart health prognosis. Similarly, another woman shared that she had only one heart scan and no other follow-ups in the 7 years since her secondary breast cancer diagnosis, despite having developed cardiac issues. She felt unsupported, discriminated against, and wondered whether the lack of monitoring was a measured clinical decision:

*‘… perhaps my oncologist thinks it would be better if I didn’t know and if I was gonna die and have a heart problem, that would be better than dying of secondary breast cancer… he would probably feel that that would be kinder for you to just be able to have a heart attack and die rather than go through all that. I know that sounds really random but that’s the kind of intuition that I have on it.’ [Interview 10.]*

Whilst some women reported discussing their heart scan results with their oncologist, many said that their heart scan results were never discussed with them. Those who received feedback said this would normally be very general, most frequently given by sonographers or nurses, less frequently from oncologists, and never from cardiology specialists. The general feedback would simply inform them that it was safe for them to carry on with Trastuzumab treatment:

*‘… the guy that was doing them [heart scans] literally said I can’t see any issues if there is anything that they want to discuss someone to be in touch.’ [Interview 1]*

Most of the women struggled to ask about scan results due to short appointment duration. Overall, most expressed that they would have liked more detailed discussions of their scan results throughout their treatment.

#### Inadequate long-term cardiac health monitoring

All women reported that, as far as they were aware, they were not receiving any longer-term follow-up on their heart function following their Trastuzumab treatment. A majority expressed worry and uncertainty as to their cardiac health, unsure whether to expect the damage to persist or to appear later in the future:

*‘…one thing don’t really know is going forward if it can cause any damage later on.’ [Interview 2]*

Likewise, some women who were on medication to support their heart function at the time of interview expressed uncertainty about their general long-term CD management plan:

*‘Do I need to be on them? Do you keep me on them? What do you do? Nobody’s had a discussion with me.’ *[10]

The women seemed overall unsure as to whether their heart health is being followed up on by any service after Trastuzumab treatment. For example, one woman who finished her treatment reported not having any regular follow-ups with an oncologist or cardiologist for up to 3 years. Another woman with pre-existing cardiac issues who also experienced heart function reduction during her Trastuzumab treatment reported that it has been over 6 years since any professional has commented on heart function during appointments. She described being given some information on heart function recovery almost incidentally:

*‘This is the first time like I say in 6–7 years that they’ve come back and sort of… automatically say that I’ve got a reduced function in the top left ventricle of my heart and I think it was during a consultation somebody said, you know that it can repair itself. And it it’s-it’s literally been that long for somebody to pick up on any sort of review.’ [Interview 11]*

### Theme 3: Knowledge on cardiac damage risks of Trastuzumab

#### Overwhelmed with information

All women reported that side effect information including CD risk information was normally presented to them at the initial or pre-treatment appointment with oncologist. This would usually be in written form, a leaflet or as part of the treatment consent form. Most women found it very difficult to process and take on board the amount of information, especially that it was normally presented to them in one go shortly after diagnosis:

*‘… far too much information to take in at one point... talking about having a mastectomy and chemotherapy and radiotherapy and hormone therapy and also the Herceptin therapy and I think it was all too much really for a normal human being to take on board.’ [Interview 13]*

One woman asked her husband to help take notes at the appointment, but even with his assistance, tracking the volume and content of information was very challenging. This struggle made some feel guilty, as if it was their ‘fault’ for not being well informed about CD risks, perhaps because they didn’t ask questions or read all the side-effects leaflets. Overall, women highlighted how ‘scary’ and overwhelming the diagnosis and start of treatment were, making it difficult to process information and make decisions. Many spoke about feeling emotionally vulnerable during treatment, expressing their complete trust and reliance on the medical professionals due to a lack of personal knowledge and experience:

*‘When you’re first diagnosed, you…you just trust the professionals that are around you because you don’t have any other knowledge or any other experience.’ [Interview 3]*

#### Unaware of cardiac damage signs

Overall, women linked a range of side effects to Trastuzumab, with severity and onset varying widely. Some experienced fatigue, shortness of breath, digestive issues, and headaches, whilst others reported no significant side-effects. Distinguishing which treatment caused which side-effect was often difficult, especially with pre-existing conditions or mixed regimens. Several women developed a notable decline in heart function during Trastuzumab treatment. It was defined by reduced left ventricular ejection fraction (LVEF), for some progressing to congestive heart failure, occasionally leading to a Trastuzumab treatment break. Heart function reduction was typically detected via imaging. Importantly, those who developed symptomatic cardiac dysfunction normally did not know which symptoms to monitor beforehand and were generally unaware of their condition:

*‘I did feel tired and maybe, you know… I did notice that my fitness levels weren’t as great, but apart from that I didn’t really notice anything anyway’ [Interview 13]*

One woman was told during a routine heart scan that she cannot have her next Trastuzumab injection because she had heart failure. She described experiencing rather severe CD symptoms at the time and feeling unwell for ‘quite a long period’ without suspecting she was in heart failure:

*‘I was really, really fatigued and I was falling asleep in the daytime whilst I was trying to work … I was very, very breathless. I couldn’t stand to walk across the room… I couldn’t stand to make myself a cup of tea because I was too fatigued and weak. I would need to sit down straight away.’ [Interview 3]*

This woman experienced co-morbid hypothyroidism, complicating diagnosis. Nonetheless, she felt understandably ‘frustrated’ by the delay in linking her symptoms to Trastuzumab. Several women with limited access to services, e.g. geographically and less knowledge of cardiac issues, said they wished for clearer information about CD symptoms and management. They believed better awareness would have prompted earlier advice-seeking, reducing potential distress and complications:

*‘…being more aware of what things to look out for would have made a difference as in I would have known what to look out for…’ [Interview 1]*

*‘… It might have made me more aware and therefore maybe do some of my own sort of research and educating myself… if I’d have known that information, I then could have then approached people.’ [Interview 3]*

#### Ill-informed about cardiac damage risks

Overall, except for a couple of women, including those with previous medical or healthcare background, most stated that they did not feel well informed about CD risks of Trastuzumab prior to or during their treatment. Many women said they felt ‘not at all’ well informed. They felt that the CD risks were not clearly presented or highlighted to them in the context of their treatment information package:

*‘… I think the risks are mentioned, but they’re not…. they don’t go into great detail about them…’ [Interview 12]*

Importantly, most women, especially those without a prior history of cardiovascular disease, said they would struggle to distinguish between various symptoms and side effects, which can be shared between cancer and other conditions. This highlights the difficulty in understanding and assessing their own CD risks:

*‘So how do you distinguish one from the other and I think patients are left not knowing how to distinguish one from the other.’ [Interview 10]*

In addition to a perceived lack of clarity, several women felt that CD risks were not presented as sufficiently ‘serious’ by professionals or in information leaflets. Most received ‘generic’ CD risk details in leaflets or documents, which were often misplaced or lost among the many papers given at diagnosis. A few noted that many breast cancer patients may struggle to read these leaflets, either due to literacy barriers or simply due to feeling overwhelmed during the diagnosis and treatment period. There was a strong desire for information tailored to personal CD risk, especially for those with pre-existing or family risks. Even when CD risk information was provided, most women felt it lacked personal relevance and detail:

*‘I would definitely wanted more detail and more detail about me and my risk…’ [Interview 7]*

With insufficient accessible CD risk information, many misjudged the seriousness of risks. Only a few actively sought information, and a few of those women stated that they have learned more about CD risks of Trastuzumab through personal research or only in case they took their own initiative to ask questions:

*‘I wouldn’t have been as informed if I hadn’t have been proactive, I think in gathering information.’ [Interview 7]*

*‘You’re just left in limbo… It’s only stuff I’ve found out myself. I didn’t know if I you know, if I decided to go for a nice walk with the dog was…was I more at risk of having a heart attack.’ [Interview 3]*

One woman who had been receiving Herceptin for the last 15 years as part of her treatment for her primary and secondary diagnoses said that she had a first-degree family history of CD, including heart failure, yet she cannot recall being asked about the family history of heart disease:

*‘… there are heart problems in my family. Both my dad had heart failure and my mum had some heart problems… I don’t remember being asked about that…’ [Interview 14]*

#### Involve patients to improve patient experience

Most of the women described it as important to be more aware of what was happening to them during treatment, as they were either not inclined to ask or did not know what to ask medical professionals. This included having more discussion of results, education on cardiac side-effects, as well as an invitation to ask questions. A couple of women described how being less informed or engaged by professionals made them feel more passive and not listened to:

*‘Um, and actually I had horrific side effects to Herceptin, and nobody would listen to me… It was more or less a case of all that’s unusual and that was it. Nobody seemed to care.’ [Interview 11]*

*‘… but you know you’ve got to learn from your patients experiences um, and it’s not just about giving people drugs.’ [Interview 8]*

Some women shared that being actively involved in their treatment, even in small ways, improved their patient experience. For example, collaborative episodes such as staff affirming patient’s knowledge of treatment/procedures, offering them to choose Trastuzumab injection site, or letting them warm the vial themselves in their hands made women feel empowerment making the treatment process less painful and more manageable:

*‘I literally I warmed the vile whilst everything else is getting done and I got to pick which leg, you know, alternated which leg it was going in… Yeah, it’s a little bit of control…its little bit of control, isn’t it?’ [Interview 4]*

Through their own and peer experience, the women saw the need for more support and advocacy, especially for those who are more vulnerable or less able to advocate for themselves, including those with secondary breast cancer:

*‘… some people are throwing themselves off a bridge because they don’t have the support.… when you have secondary cancer, this is…. this is how left you are, you’re abandoned, you don’t get follow-ups, you don’t have the interventions, you don’t have the support. I’m basically left to-to manage my own disease. That’s how I feel.’ [Interview 10]*

## Discussion

To our knowledge, our study is the first to investigate patient experience of Trastuzumab and knowledge of cardiac damage risk because of Trastuzumab using a qualitative approach in women who had undergone or were receiving Trastuzumab for breast cancer at the time of study. The thematic analysis of our responses revealed three main latent themes. These reflected poor patient knowledge and awareness of cardiac damage prior to starting Trastuzumab treatment, inconsistent monitoring of cardiac health during and beyond treatment, and a lack of awareness of symptoms of cardiac damage due to Trastuzumab treatment.

Without exception, at the outset of treatment with Trastuzumab, all women stated that treating the cancer was the priority and that the risks of cardiac damage had little to no impact on their decision. When Trastuzumab treatment caused evidence of heart failure and treatment was interrupted, women were anxious to resume Trastuzumab after a break. Women described how they believed Trastuzumab was the ‘wonder drug’, the ‘best thing’, and how grateful they were for the treatment. Women believed that the cancer would ‘finish them off’ but that cardiac damage would be ‘fixed’. For some women who were opposed to chemotherapy as part of their treatment regimen, they would happily agree to receiving chemotherapy if they were promised Trastuzumab. Women’s perseverance in ensuring the receipt of Trastuzumab is a testament to the existing bias in favour of the benefits of this drug compared to the scarcity of knowledge on the risks associated with Trastuzumab treatment [10]. This has implications for increasing the vulnerability of patients with pre-existing heart conditions as well as those with prior cardiac damage for longer term decline in heart function and possible cardiovascular disease [20].

Our study revealed that the risk/benefit imbalance in knowledge of Trastuzumab effects did not come without psychological consequences. The interviews revealed that some women felt guilty for not asking the ‘right’ and ‘relevant’ questions regarding possible cardiac damage effects of Trastuzumab either prior to or during treatment, and they felt that it was somehow their fault if they had experienced cardiac malfunctioning. Feelings of guilt and blame are strong dispositional factors for poor self-confidence and self-esteem, increasing the risk of depression [21]. In a recent large meta-analysis [22] anxiety and depression in women diagnosed with breast cancer were found to significantly increase the risk of mortality from this disease by up to 30%. In related research, women who had poorer self-ratings of their health also had lower predicted survival outcomes [23]. These findings clearly indicate that the psychological health of breast cancer survivors can influence clinical outcomes with implications for physical health.

Our findings showed women were poorly educated for signs and symptoms of Trastuzumab side effects, generally as well as specifically, for cardiac damage, which can reflect poor doctor-patient communication. Women reported that they often gathered information from external sources such as support groups or the internet to learn about Trastuzumab’s specific side effects. There was a consensus that leaflets on risks of cardiac malfunction provided at the time of diagnosis were not helpful as the amount of information to be processed was overwhelming and distressing. Similarly, as participants reflected, leaflets or booklets could have been misplaced or piled with other information handed out routinely. This finding suggests that a more direct and clear mode of communication with an oncologist or breast cancer nurse regarding awareness of signs and symptoms of Trastuzumab treatment is necessary [10].

Women felt reassured that the ongoing monitoring of heart function would detect cardiac damage from Trastuzumab. However, the responses indicated a large level of inconsistency in the monitoring of heart function, with some women receiving sporadic scans across treatment or no scans beyond treatment even if they had experienced cardiac damage during treatment. One woman mentioned that she accidentally discovered about her cardiac damage through a phone call many years after Trastuzumab treatment had ended. Many women had not seen a cardiologist or were unaware of the relevance or input of a cardiologist in their treatment plan. Furthermore, the details of electrocardiogram assessments and measurements were not communicated, either by oncologists, nurses, or cardiologists. In fact, the understanding was that if a problem was noted, the patient would be informed and Herceptin would be discontinued. This increased patient anxiety about possible treatment ineffectiveness as well as fear of cancer recurrence. Growing research repeatedly emphasises the importance of patient monitoring during and beyond Trastuzumab treatment [24–26].

Further complications were highlighted for those with a history of cardiac disease. Some of our participants could not remember if they had been questioned about a family history of cardiac damage prior to Trastuzumab treatment. Specifically, one of our participants who had been taking Trastuzumab for numerous years following her primary and metastatic diagnosis had first-degree family history of cardiac damage and did not recall if she was asked about family history of cardiac damage. Similar anxieties were noted for those developing cardiac malfunction, who were not monitored during or after treatment. In fact, it was possible to receive as few as only two scans during an 18-month treatment of Trastuzumab. A patient with metastatic breast cancer with additional cardiac complications had received only a couple of scans during a 7-year follow-up. Whilst different treatment regimens regarding cardiac function monitoring are possible, patients require an understanding of their treatment plan, and a clear communication strategy is needed with their oncologist to manage expectations accordingly [27, 28].

Many women reported feeling unsupported, anxious, and discriminated against, especially if they had metastatic breast cancer. The pressing problem arose for those with cardiac complications due to Trastuzumab treatment who were not followed up once treatment had ended. Women raised concerns regarding a lack of interest from oncologists in their cardiac health and were uncertain about what measures or medications they should or could be taking to prevent further damage. One woman with metastatic breast cancer who had suffered damage to the left ventricle of the heart because of Trastuzumab treatment described discovering this damage by accident 6 years after having no contact with her oncologist.

## Conclusions

This study has shown that awareness and knowledge of Trastuzumab cardiotoxicity and associated symptoms needs to be raised, and risks of cardiac damage communicated with patients undergoing treatment for breast cancer. Inconsistencies in patient monitoring throughout and beyond treatment are a cause for concern. Poor doctor-patient communication and disparities in procedures can increase worries and anxieties, with some patients feeling let down by their medical teams. Participants in this study expressed a desire to be informed and as well as work in collaboration with their oncologists to support a personalised treatment option. Knowledge of personalised risk and benefit can improve the management of symptoms as well as patient adherence to the treatment regimen.

## Limitations and future directions

Future research should follow up the current study with a large-scale mixed modality design increasing the opportunity for respondents without digital access to partake in such studies. This will reduce bias and increase representation for further insight into the experiences of women treated with Trastuzumab. Our sample overrepresented white educated British women, which can limit generalisability of our findings to the wider breast cancer survivor population. As such, our study may not capture perspectives of women from other backgrounds. Future studies should make active efforts to encourage interest and participation of women from more diverse socioeconomic, ethnic, and cultural backgrounds. This can be achieved via increasing outreach to minority communities, using targeted adverts, and by adapting materials and conducting interviews in other languages. Finally, future qualitative studies should consider the impact of Trastuzumab-related cardiac damage risks on quality of life in different age groups to help tailor future support and advice. Our study did not capture the voices of younger (< 40 years old) or older breast cancer survivors (> 65 years old) who may have specific needs and concerns regarding Trastuzumab effects on cardiac damage.

## Data Availability

The data supporting the findings of this study is not publicly available as participants did not agree to their data to be shared publicly. Participants of this study did, however, provide consent for the result to be written up for publication.
